# Diagnostic value of protein induced by vitamin K absence (PIVKAII) and hepatoma-specific band of serum gamma-glutamyl transferase (GGTII) as hepatocellular carcinoma markers complementary to *α*-fetoprotein

**DOI:** 10.1038/sj.bjc.6601018

**Published:** 2003-06-10

**Authors:** R Cui, J He, F Zhang, B Wang, H Ding, H Shen, Y Li, X Chen

**Affiliations:** 1 Liver Research Center, Beijing Friendship Hospital, Capital University of Medical Science, Beijing 100050, China; 2 Medical Analysis Department, First Educational Hospital, Lanzhou Medical College, Lanzhou, Gansu 730000, China; 3 Beijing Youan Hospital, Capital University of Medical Science, Beijing 100050, China

**Keywords:** protein induced by vitamin K absence or antagonist II, *α*-fetoprotein, hepatoma-specific band of serum gamma-glutamyl transferase, hepatocellular carcinoma, diagnosis

## Abstract

Serum protein induced by vitamin K absence or antagonist II (PIVKAII), hepatoma-specific band of serum gamma-glutamyl transferase (GGTII), and *α*-fetoprotein (AFP) levels were determined in 120 patients with hepatocellular carcinoma (HCC) and 90 patients with cirrhosis. The mean serum concentration of PIVKAII in HCC patients was higher than that in cirrhotic patients. A total of 53.3% of patients (64 out of 120) with HCC had PIVKAII levels above 40 mAU ml^−1^. However, only 13 patients with cirrhosis had higher PIVKA II levels. Of 32 small HCC patients, 13 (40.6%) had PIVKAII values above 40 mAU ml^−1^. An increased concentration of AFP (i.e. 20 ng ml^−1^) was observed in 70 out of 120 (58.3%) patients with HCC and in 33 out of 90 (36.7%) patients with cirrhosis. Positive GGTII was found in 74.0% (89 out of 120) cases of HCC (sensitivity), in 16 of 90 cases of cirrhosis, and 14 of 32 (43.8%) small HCC patients had GGTII positive. No significant correlation was found between serum levels of AFP and PIVKAII. Combining the information from PIVKAII, AFP, and GGTII significantly increases the sensitivity over AFP alone. PIVKAII and GGTII are useful tumour markers complementary to AFP for diagnosis of HCC.

Hepatocellular carcinoma (HCC) is one of the most prevalent causes of death in the world. As the majority of HCCs develop from cirrhotic livers, particularly the posthepatitic or macronodular variety, patients with cirrhosis are recommended to undergo regular examinations for early detection of possible HCC (Okuda, 1986). Measurements of protein induced by vitamin K absence or antagonist-II (PIVKAII) and *α*-fetoprotein (AFP) concentration, along with several imaging modalities, have been used widely in Japan and North America (Liebman *et al*, 1984; Okuda *et al*, 1987; Fujiyama *et al*, 1988; Hattori *et al*, 1988). Although PIVKAII is a tumour marker complementary to AFP for diagnosis of HCC, it has not been used in China. We have determined PIVKAII levels in 60 patients with HCC and 30 patients with cirrhosis in China in order to determine the usefulness of PIVKAII for diagnosing HCC (Cui *et al*, 2002). We concluded that PIVKAII is a useful marker for early diagnosis of HCC and may improve the sensitivity of diagnosis when combined with AFP.

In 1984, Liebman *et al* first reported a specific increase of plasma PIVKAII in HCC patients. Since then, an increasing number of studies report that PIVKAII is a useful tumour marker complementary to AFP in detecting HCC (Liebman *et al*, 1984; Taketa *et al*, 1990; Sato *et al*, 1993). Serum PIVKAII has attracted attention because of its very high specificity and lack of correlation with serum AFP levels.

In 1965, Kokot and Kuska separated the serum gamma-glutamyl transferase (EC 2.3.3.3, GGT) into three to four bands by means of paper electrophoresis (Kokot and Kuska, 1965). Since then, other methods have been used, that is, separation of GGT bands by means of starch gel (Orlowski and Szczeklik, 1967), cellulose acetate (Hitoi *et al*, 1984), agarose gel (Hetland *et al*, 1975), polyacrylamide gel electrophoresis (Kojima *et al*, 1980; Suzuki *et al*, 1981; Sawabu *et al*, 1983; Kew *et al*, 1984), and polyacrylamide stage gel plate (Xu *et al*, 1985). The polyacrylamide stage gel plate method allows detecting positivity up to 90% in hepatoma patients (Xu *et al*, 1985).

In this study, we have determined serum PIVKAII and GGTII levels in patients with HCC or cirrhosis using ED036 kit and polyacrylamide stage gel plate, respectively, in order to assess the diagnostic values of PIVKAII, GGTII combined with AFP for clinical application.

## MATERIALS AND METHODS

### Patients

Serum PIVKAII and AFP levels and GGTII activity were determined in 90 patients with cirrhosis and 120 patients with HCC (their clinical characteristics are summarised in Table 1

**Table 1:** Clinical Features of patients with HCC and cirrhosis in the present study

**Feature**	**HCC**	**Cirrhosis**
Median age (years) (range)	59 (32–74)	56 (32–84)
Male/female ratio	2.7	2.1
Complication of cirrhosis	54.2% (65/120)	72.2% (65/90)
Positive for HBsAg	80.8% (97/120)	92.2% (83/90)
Positive for anti-HCV	0	1
Positive for both HBsAg and anti-HCV	0	0
Negative for both HBsAg and anti-HCV	17.5% (23/120)	8.9% (8/90)

HCC=hepatocellular carcinoma; HBsAg=hepatitis B surface antigen; anti-HCV= antibody to hepatitis C virus.

). In patients with cirrhosis, HCC was ruled out on the basis of imaging examinations including sonography and computed tomography (CT) performed on a regular basis. Also, cirrhotic patients who developed HCC within 1 year from getting serum were excluded. In all, 58% (70 out of 120) of HCC patients were diagnosed by fine-needle biopsy under the guidance of ultrasonography, and in 16% (19), the diagnosis was confirmed after surgery. Ultrasonography, CT, magnetic resonance imaging, and selective celiac angiography diagnosed the remaining patients (26%, 31 out of 120). Computed axial tomography (CAT) scan or celiac angiogram estimated the two-dimensional size of the tumour included in this study. Medical history, HBV and HCV infections, and liver functions (aspartate transaminase (AST), alanine transaminase (ALT), GGT, alkaline phosphatase, albumin and bilirubin levels of serum) were recorded, and vitamin K and antibiotics were used. Patients with vitamin K and antibiotic use in the recent 3 months, with a hemoglobin levels under 490 mg dl^−1^ and free bilirubin concentrations up to 27 mg dl^−1^ or conjugated bilirubin concentrations up to 22 mg dl^−1^ were excluded from this study. Venous blood samples of the patients were obtained with a syringe containing a 3.8% sodium citrate solution (9 : 1). The samples were immediately centrifuged, and the serum was stored at −20°C until the analysis. Informed consent was obtained from each patient included in the study. The study protocol conforms to the ethical guidelines of the 1975 Declaration of Helsinki as reflected in *a priori* approval by the human research committee of Beijing Friendship Hospital.

### Assay to determine serum PIVKAII concentrations

The serum PIVKAII concentrations were measured by the sensitive enzyme immunoassay (EIA) (ED-036 kit, Eisai Laboratory, Tokyo, Japan) according to the manufacturer's instructions. The principle of measurement is solid-phase, sandwich EIA. Briefly, using solidified anti-PIVKAII monoclonal antibody, PIVKAII in the specimen is allowed to react with enzyme-labelled antihuman prothrombin antibody. When substrate solution is added to the reaction product, the enzyme substrate in the substrate solution decomposes, allowing the colouring agent to develop a colour. The content of PIVKAII in the specimen is determined from the absorption of the coloured solution. The cutoff value for the sensitive EIA was set at 40 mAU ml^−1^ according to the manufacturer's instructions.

### Serum GGTII

GGT activity assay was carried out on a vertical slab stage polyacrylamide gel as described by Xu *et al* (1985). In brief, there are three layers of separation gels: 15, 10, and 7.5% from the bottom upward. The stacking polyacrylamide gel concentration is 4%. A measure of 20 *μ*l of serum mixed with an equal volume of Laemmli buffer was loaded on the gel. After electrophoresis, the gel plate was incubated at 37°C for 60 min with a substrate mixture containing 0.82 mmol l^−1^ glutamyl-*p*-nitroanilide, 100 mmol l^−1^ tris, 100 mmol l^−1^ glycyl glycine, and 3.4 mmol l^−1^ N-(1-naphthyl)-ethylenediamine dihydrochloride. Finally, the gel plate was immersed in a solution containing 10 g of trichloroacetic acid and 25 g of glycerol for every 100 ml, and within a few minutes the red bands corresponding to GGT appeared.

### Serum AFP concentration

The serum concentration of AFP was determined by electrochemiluminescence immunoassay (Roche, Elecsys 1010/2010 Systems) according to the manufacturer's instructions. The cutoff level was fixed at 20 ng ml^−1^.

### Statistical analysis

Values are presented as mean ±s.d. The results were analysed with the *χ*^2^ test, F-test, and Spearman's test.

## RESULTS

### Serum levels of PIVKAII

The mean values of serum PIVKAII in 90 cirrhotic patients and 120 HCC patients were 18.4±30.6 and 644.4±146121 mAU ml^−1^, respectively. There was no significant difference between the mean values of PIVKAII in male and female HCC or cirrhotic patients (*P*<0.05). There was a statistically significant difference between the mean values of PIVKAII in HCC patients and cirrhosis patients (*P*<0.001).

In all, 13 out of 90 (14%) patients with cirrhosis, and 64 of 120 (53%) with HCC had PIVKAII levels above the detection limit of 40 mAU ml^−1^. The overall accuracy, sensitivity, specificity, and positive and negative predictive values for the usefulness in the diagnosis of patients with HCC are given in Table 2

**Table 2:** Diagnostic values of PIVKAII for the detection of HCC

	**PIVKAII**	**GGTII**
Overall accuracy	67.1% ((64+77)/(90+120))	77.6% ((89+74)/(90+120))
Sensitivity	53.3% (64/120)	74.2% (89/120)
Specificity	85.6% (77/90)	82.2% (74/90)
Positive predictive value	83.1% (64/(64+13))	84.8% (89/(89+16))
Negative predictive value	57.9% (56/(56+77))	70.5% (74/(31+74)

Overall accuracy=(TP+TN)/(TP+FP+TN+FN); Sensitivity=TP/(TP+FN); Specificity=TN/(FP+TN); Positive predictive value=TP/(TP+FP); Negative predictive value=TN/(FN+TN); TP=true positive; TN=true negative; FP=false positive; FN=false negative.

. No correlation was found between serum levels of PIVKAII and liver functions (AST, ALT, GGT, alkaline phosphatase, albumin, and bilirubin levels in serum, data not shown).

### GGTII in patients with HCC and cirrhosis

Positive GGTII was found in 89 of 120 (74%) patients with HCC and in 16 of 90 (18%) patients with cirrhosis. The overall accuracy, sensitivity, specificity, and positive and negative predictive values for the usefulness in the diagnosis of patients with HCC are given in Table 2. No correlation was found between serum GGTII and liver functions (AST, ALT, GGT, alkaline phosphatase, albumin and bilirubin levels of serum, data not shown).

### Correlation between tumour size and PIVKAII level

**Figure 1:**
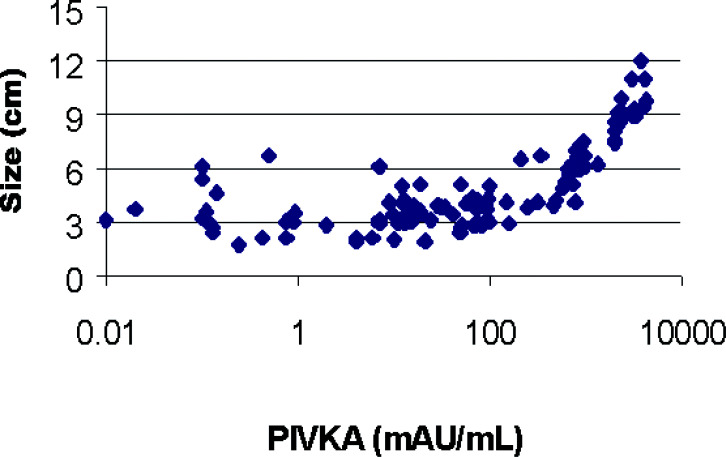
Correlation between tumour size and PIVKAII level.

The correlation of tumour size and PIVKAII level in 120 HCC cases is shown in Figure 1

. Overall, patients with bigger tumours tend to have higher levels of PIVKAII.

### Relation between serum levels of AFP, PIVKAII, and positive GGTII

An AFP concentration above 20 ng ml^−1^ was observed in 70 of 120 patients with HCC (58.3%) and in 33 of 90 patients with cirrhosis (36.7%). The remaining 50 patients with HCC had AFP concentrations of less than 20 ng ml^−1^, and 24 of 50 patients (48.0%) had elevated concentrations of PIVKAII. No significant correlation was found between serum levels of AFP and PIVKAII in 120 HCC patients (*r*_s_=0.106, *P*=0.249). Of 50 HCC patients, 31 (62.0%) without increased AFP had been GGTII positive. No significant correlation was found between serum levels of AFP and PIVKAII in 120 HCC patients.

Combining the information on PIVKAII and AFP levels significantly increases the sensitivity of diagnosis over AFP alone; it showed an increase of approximately 20% above AFP alone and 25% above PIVKAII alone. Also, combining the information from AFP levels and GGTII positive significantly increases the sensitivity of diagnosis above AFP alone; it showed an increase of approximately 25.9% above AFP alone and 10.2% above GGTII alone. Combining the information from PIVKAII and AFP levels, and GGTII positive significantly increases the sensitivity of diagnosis above AFP alone; it showed an increase of approximately 29.2% above AFP alone, 34.2% above PIVKAII alone, and 13.5% above GGTII alone (Table 3

**Table 3:** Sensitivity and specificity of serum PIVKAII and AFP in patients with HCC and small sized HCC

	**PIVKA II**		**AFP**		**GGT II**	
	**HCC**	**Small-sized HCC**	**HCC**	**Small-sized HCC**	**HCC**	**Small-sized HCC**
Sensitivity (%)	53.3	40.6	58.3	43.8	74.0	43.8
Specificity (%)	85.6	85.6	63.3	63.3	82.2	82.2
	**PIVKA II+AFP**		**AFP+GGT II**		**PIVKA II+AFP+GGT II**	
	**HCC**	**Small-sized HCC**	**HCC**	**Small-sized HCC**	**HCC**	**Small-sized HCC**
	78.3	59.4	84.2	59.4	87.5	71.9
	58.9	58.9	55.6	55.6	53.3	53.3

PIVKAII=protein induced by vitamin K absence or antagonist II; AFP: *α*-fetoprotein; PIVKAII+AFP=PIVKAII and/or AFP positive; HCC=hepatocellular carcinoma.

).

For HCC patients with tumours approximately less than 3 cm in dimension, combining the information from PIVKAII and AFP levels significantly increases the sensitivity of diagnosis above AFP alone also. It showed an increase of approximately 15.6% above AFP alone and 18.8% above PIVKAII alone (Table 3). Combining the information from PIVKAII and AFP levels, and GGTII positive significantly increases the sensitivity of diagnosis above AFP alone; it showed an increase of approximately 28.1% above AFP alone, 31.3% above PIVKAII alone, and 28.1% above GGTII alone (Table 3).

## DISCUSSION

The diagnostic value of elevated AFP levels has been established, but increased levels of AFP are also observed in acute and chronic liver diseases as well as in other malignancies. Moreover, there are false-negative results ranging between 10 and 50% depending on ethnic and geographical variations and the techniques employed. It is thus necessary to combine with markers for diagnosis of HCC in a clinical setting. PIVKAII and GGTII are useful and early diagnostic markers for HCC.

**Figure 2:**
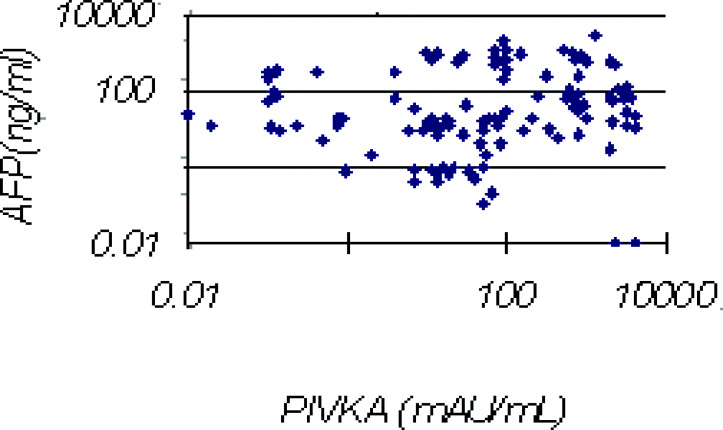
Relation between PIVKAII and AFP levels in the 120 patients with HCC.

The amino-terminal glutamic acid residues of prothrombin precursor undergo carboxylation in the liver, resulting in normal prothrombin containing 10 gamma-carboxy-glutamic acid residues. The absence of vitamin K or the presence of its antagonist inhibiting the vitamin K-dependent carboxylase activity results in the secretion of immature prothrombin into the blood that lacks gamma-carboxy-glutamic acid residues (i.e. PIVKAII). Although the precise mechanisms of PIVKAII production in HCC are not fully understood, PIVKAII is now widely accepted as a tumour marker for HCC in Japan (Ishii *et al*, 2000).

In 1984, Liebman *et al* measured abnormal prothrombin by a competition radioimmunoassay using a conventional polyclonal antibody in the serum of 76 HCC cases and reported increased abnormal prothrombin in 69 (91%) cases; the level of these cases exceeded 300 ng ml^−1^ in 51 (67%) cases. They also described that only three out of 28 cases (11%) with chronic active hepatitis showed an increased abnormal prothrombin level. Together, the assay for abnormal prothrombin and AFP identified 84% of 76 cases with HCC. Thus, they concluded that these two tests are complementary tumour markers in this disease (Liebman *et al*, 1984). Since then, there have been many publications about the usefulness of PIVKAII in the diagnosis of patients with HCC. The sensitivity of PIVKAII in the diagnosis of patients with HCC had been about 55% using a conventional kit (EI-test mono-P-II kit, Eisai Laboratory) before 1998. Several studies on the contrasting sensitivity kit and the conventional kit confirmed that the new kit is more sensitive than the conventional one in diagnosing HCC patients after 1998 (Mita *et al*, 1998; Nomura *et al*, 1999). The sensitivity is about 65% in patients diagnosed with HCC (Mita *et al*, 1998). We measured the serum level of PIVKAII using the sensitive kit in this study. There have been several publications about the usefulness of PIVKAII in monitoring recurrence in patients with HCC, as a prognostic marker in HCC, and as a marker of post-transplant graft function (Nakao *et al*, 1995; Taketoshi *et al*, 1995; Aoyagi *et al*, 1996; Suehiro *et al*, 1998).

In 1965, Kokot and Kuska separated the serum gamma-glutamyl transferase (EC 2.3.3.3, GGT) into three to four bands by means of paper electrophoresis (Kokot and Kuska, 1965). Other researchers used polyacrylamide gel gradient electrophoresis to fractionate one to three bands of GGT specific for hepatoma with a positivity rate of only 27–63% (Hetland *et al*, 1975; Kojima *et al*, 1980; Sawabu *et al*, 1983; Kew *et al*, 1984). In 1985, Xu reported that they had fractionated nine to 11 activity bands of GGT adopting the method of vertical slab stage electrophoresis on polyacrylamide gel (Xu *et al*, 1985), in which GGTII was found in the sera of all patients with hepatoma. The positive rate of GGT was 90% and no correlation was observed with AFP. After 10 years of follow-up, they reported the following: (1) GGTII was positive in 90% of 90 cases with HCC and negative in most patients with acute and chronic viral hepatitis, extrahepatic tumours, in pregnant women, and in healthy controls. (2) The positive rate of GGTII assay was higher than that of alkaline phosphatase isoenzyme I (ALP AFP, and alpha 1-antitrypsin (AAT)) in 101 cases with HCC. In cases where the AFP level was greater than 50 ng ml^−1^ or less than 50 ng ml^−1^, the positive rates of GGTII were 70.8 and 75–100%, respectively. (3) Of the 14 cases of small-size HCC, the positive rate of GGTII was 78.6%, which was higher than that of AFP (50%), AAT (28.6%), and ALP I (0%). (4) Of the 62 cases that were false-positive for the GGTII assay, 24.2% developed into HCC during a follow-up of 2.1–20 months. Approximately 86.7% of subjects with persistent positivity of GGTII and 22.2% of subjects with recurrent positivity developed HCC. No patient with temporal positivity of GGTII developed HCC (Xu *et al*, 1992). Yao modified the assay procedure for the quantity detection of GGTII; however, its sensitivity is less than the procedure without quantity detection (Yao *et al*, 1998).

In this study, PIVKAII was proved to be useful for the diagnosis of HCC in China. The sensitivity, specificity, and accuracy are about 53.3, 85.6, and 67.1%, respectively, for all HCC patients. These figures were similar to those in the study of Okuda *et al* (1999) (45, 92.8, and 63.7%, respectively) but higher than those in the study of Kuromatsu *et al* (1997) (27.9, 100, and 56.1%, respectively). Most of the HCC patients in the Kuromatsu study had tumours smaller than 3 cm, whereas more than half of our HCC patients had tumours larger than 3 cm. The sensitivity is about 40.6% for small-sized HCC patients. The figures were higher than those in the study of Saitoh *et al* (1994) (24%, eight out of 34) and lower than those in the study of Mita *et al* (1998) (71%, 12 out of 17) because their criteria of small-sized HCC were different from ours. GGTII was proved to be a useful tumour marker for HCC diagnosis, although its sensitivity was less than that in Xu *et al*'s reports (90%).

Measurement of AFP, PIVKAII, and GGTII may be more advantageous than the measurement of only a single marker. In fact, in the current cases with HCC development, AFP, PIVKAII, and GGTII increase independently. The sensitivity of PIVKAII in the HCC patients without increased AFP is about 48.0% in this study. This figure was lower than the sensitivity in the study of Mita *et al* (1998) (69%). In the study of Mita *et al* (1998), the sensitivities of HCC, small-size HCC, and well-differentiated HCC were also much higher than the sensitivities in the study of Saitoh *et al* (1994) and our study. Okuda *et al* (1999), Kuromatsu *et al* (1997), and Saitoh *et al* (1994) did not report the sensitivity of PIVKAII in the HCC patients without increased AFP, so we are unable to compare the senstivities. However, Saitoh *et al* (1994) studied the correlation between AFP and PIVKAII measured by the ABC method in patients with small-sized HCC and found no apparent correlation. Kuromatsu and Okuda also detected no obvious correlation between AFP and PIVKAII measured by the revised EIA kit in their HCC patients (Kuromatsu *et al*, 1997; Okuda *et al*, 1999). The sensitivity of GGTII in patients without increased AFP was about 62%, which was less than that in Xu *et al*'s (1985) report (84.6%). All papers about GGT in the HCC diagnosis report no apparent correlation between AFP and GGTII, consistent with the results of this study. Publications on the correlation between GGTII and PIVKAII are not available. Our study showed no apparent correlation between GGTII and PIVKAII. The difference in the sensitivity of these two markers may be due to different synthetic pathways in the cell. Thus, employing these three complementary markers is useful in the diagnosis of developing HCC, as shown in this study.

In conclusion, PIVKAII and GGTII are useful tumor markers complementary to AFP for the diagnosis of HCC.
